# Relationship between B-type natriuretic peptide levels and echocardiographic indices of left ventricular filling pressures in post-cardiac surgery patients

**DOI:** 10.1186/1476-7120-7-49

**Published:** 2009-10-28

**Authors:** Alessandro Salustri, Elena Cerquetani, Mara Piccoli, Guglielmo Pastena, Alfredo Posteraro, Elisabetta Amici, Salvatore La Carrubba, Sherif Bakir, Wael Abdulrahman Al Mahmeed

**Affiliations:** 1 Institute of Cardiac Science, Sheikh Khalifa Medical City, P.O. Box 51900, Abu Dhabi, United Arab Emirates; 2 Cardiology Department, Policlinico Luigi di Liegro, Via dei Badoer 5, Roma, Italy; 3 Internal Medicine, Villa Sofia Whitaker Hospital, Piazza Salerno 1, Palermo, Italy

## Abstract

**Background:**

B-type natriuretic peptide (BNP) is increased in post-cardiac surgery patients, however the mechanisms underlying BNP release are still unclear. In the current study, we aimed to assess the relationship between postoperative BNP levels and left ventricular filling pressures in post-cardiac surgery patients.

**Methods:**

We prospectively enrolled 134 consecutive patients referred to our Center 8 ± 5 days after cardiac surgery. BNP was sampled at hospital admission and related to the following echocardiographic parameters: left ventricular (LV) diastolic volume (DV), LV systolic volume (SV), LV ejection fraction (EF), LV mass, relative wall thickness (RWT), indexed left atrial volume (_i_LAV), mitral inflow E/A ratio, mitral E wave deceleration time (DT), ratio of the transmitral E wave to the Doppler tissue early mitral annulus velocity (E/E').

**Results:**

A total of 124 patients had both BNP and echocardiographic data. The BNP values were significantly elevated (mean 353 ± 356 pg/ml), with normal value in only 17 patients (13.7%). Mean LVEF was 59 ± 10% (LVEF ≥50% in 108 pts). There was no relationship between BNP and LVEF (p = 0.11), LVDV (p = 0.88), LVSV (p = 0.50), E/A (p = 0.77), DT (p = 0.33) or RWT (p = 0.50). In contrast, BNP was directly related to E/E' (p < 0.001), LV mass (p = 0.006) and _i_LAV (p = 0.026). At multivariable regression analysis, age and E/E' were the only independent predictors of BNP levels.

**Conclusion:**

In post-cardiac surgery patients with overall preserved LV systolic function, the significant increase in BNP levels is related to E/E', an echocardiographic parameter of elevated LV filling pressures which indicates left atrial pressure as a major determinant in BNP release in this clinical setting.

## Background

B-type natriuretic peptide (BNP), a cardiac hormone synthesized by ventricular myocytes in response to left ventricular (LV) dysfunction and wall stress, has been shown to be increased in patients with cardiovascular disease such as heart failure,[1] cardiac hypertrophy,[2] and acute coronary syndromes [3]. Although in most of these situations the increase in BNP is related to systolic LV dysfunction [4], recent evidences suggest that, at least in patients with heart failure, elevated LV filling pressures may also act as a trigger for BNP release [5,6] and are likely to reduce exercise capacity [7]. More recently, high levels of BNP have also been reported early after cardiac surgery, with prognostic implications for prolonged hospital stay and 1-year mortality [8]. However, it is worth noting that in post-cardiac surgery patients, as compared to patients with heart failure, LV ejection fraction does not correlate as much as expected with BNP levels [9]. Thus, in this clinical setting, factors other than LV systolic function, such as myocardial damage during aortic cross clamping, have been advocated;[10] however the results of these studies are still controversial and the mechanisms underlying BNP release remain unclear.

We hypothesized that elevated LV filling pressures in post-cardiac surgery patients may account for the increase in plasma BNP. Thus, we designed this prospective study with the aims of assessing the relationship between postoperative BNP levels and LV filling pressures as evaluated by transthoracic two-dimensional and Doppler echocardiography.

## Methods

We prospectively enrolled 134 consecutive in-hospital patients admitted to our Centre for a cardiac rehabilitation program 8 ± 5 days after a cardiac surgery procedure. No patients required urgent cardiac surgery due to acute myocardial infarction or acute heart failure. Patients in haemodialysis were excluded. All patients were clinically stable and followed the medical therapy prescribed by the referring physicians.

Participants in the study had complete data on age, sex, diabetes mellitus (glucose level >126 mg/dl or hypoglicemic drugs), renal insufficiency (serum creatinine value ≥1.5 mg/dl), type of cardiac surgery, and length of hospitalization. The investigation conforms with the principles outlined in the Declaration of Helsinki. The hospital institutional review board approved the study and written informed consent to participate in the study was obtained from all patients.

### BNP measurements

Blood samples (5 ml) were obtained 24 hours after patient admission. In all patients, blood was sampled from an antecubital vein after 15 minutes of supine rest and collected into a sampling tube containing potassium EDTA. Samples were analyzed for BNP levels using a microparticle enzyme immunoassay (Abbott Laboratories, Abbott Park, Illinois, USA). The precision and sensitivity of this kit have been previously described by Rawlins et al [11].

### Echocardiography

All the patients underwent a comprehensive echocardiographic study within 24 hours after admission at our Center using a commercially available ultrasound equipment (Philips Sonos 5500, Andover, Massachusetts, USA). All measurements were made according to the recommendations of the American Society of Echocardiography/European Association of Echocardiography [12].

The left ventricular (LV) assessment included measurement of the diastolic (DV) and systolic (SV) volumes with derived ejection fraction (LVEF) using the modified biplane Simpson's method, mass, and relative wall thickness (RWT). Left atrial volume was derived using the modified biplane Simpson's method and the value was indexed by body surface area. Mitral inflow was assessed from the apical 4-chamber view using pulsed-wave Doppler. Early-to-late diastolic velocities (E/A) ratio was calculated, as well as the deceleration time (DT) of the E wave. The early diastolic velocities at the septal and lateral mitral annulus were obtained from the apical 4-chamber view using Doppler tissue imaging, and the values were averaged (E'). LV filling pressure was estimated by the E/E' ratio [13].

### Statistics

All analyses were performed by using SPSS for Windows (version 12.0.1, Chicago Ill.). Continuous variables are expressed as mean ± SD. Categorical variables are expressed as number of subjects and percentages. Since plasma concentrations of BNP did not follow a Gaussian distribution, the values were log-transformed before statistical analysis. We used the independent Student's t test for univariate comparison of log-transformed BNP [ln(BNP)] value within dichotomic variables. Correlations between continuous variables were tested by Pearson's correlation test. Multiple regression analysis was used to clarify the contribution of each independent variable to BNP having ln(BNP) as the dependent variable. Statistical significance was assumed at p < 0.05.

## Results

Ten patients were excluded because of poor acoustic windows that resulted in incomplete echocardiographic measurements. Thus, a total of 124 patients with BNP and echocardiographic data formed the final group and were included in the analysis (Table 1).

**Table 1:** Clinical characteristics of the study population.

Number of patients	124
Male, nr (%)	83 (67)

Age, mean ± SD (yrs)	67 ± 10

Days after cardiac surgery, mean ± SD	8 ± 5

Type of surgery, nr (%)	

- CABG	67 (54)

- Valve surgery	39 (31)

- CABG + valve surgery	11 (9)

- Aorta replacement	7 (6)

Renal failure, nr (%)	18 (14)

Diabetes, nr (%)	30 (24)

Medical therapy, nr (%)	

- betablockers	72 (61)

- ACE-inhibitors	81 (78)

- ARB	15 (13)

- Diuretics	92 (78)

ACE = angiotensin converting enzyme; ARB = angiotensin receptor blockers; CABG = coronary artery bypass graft.

### BNP after cardiac surgery

Plasma BNP levels were significantly elevated in the study group (range 39-2808 pg/ml; mean 353 ± 346 pg/ml) and only 17 (13.7%) patients had normal BNP levels (<100 pg/ml). After log-transformed, the mean ln(BNP) was 5.54 ± 0.8 (range 3.66-7.94). The mean value of BNP was significantly higher for women compared to men, while no differences were found according to the presence of diabetes mellitus, renal failure, or coronary artery bypass graft (see Table 2). There was a mild but significant association between BNP and patient's age (Pearson's coefficient = 0.302, p < 0.001), while length of hospital stay was not associated with BNP (Pearson's coefficient = -0.99, p = 0.273).

**Table 2:** Mean value of ln(BNP) according to female gender, presence of renal failure, diabetes mellitus, and coronary artery by-pass surgery (CABG).

	N (%)	Mean (SD)	p value
Female gender	41 (33)	5.76 (0.80)	

			0.032

Male gender	83 (67)	5.44 (0.79)	

			

			

Diabetes mellitus, yes	30 (24)	5.71 (0.77)	

0.197			

Diabetes mellitus, no	94 (76)	5.49 (0.81)	

			

			

Renal failure, yes	18 (15)	5.71 (0.96)	

0.358			

Renal failure, no	106 (85)	5.52 (0.78)	

			

			

CABG	67 (54)	5.61 (0.84)	

0.312			

Other cardiac surgery	57 (46)	5.47 (0.76)	

			

			

Total	124 (100)	5.55 (0.80)	

### BNP and echocardiographic parameters

**Figure 1:**
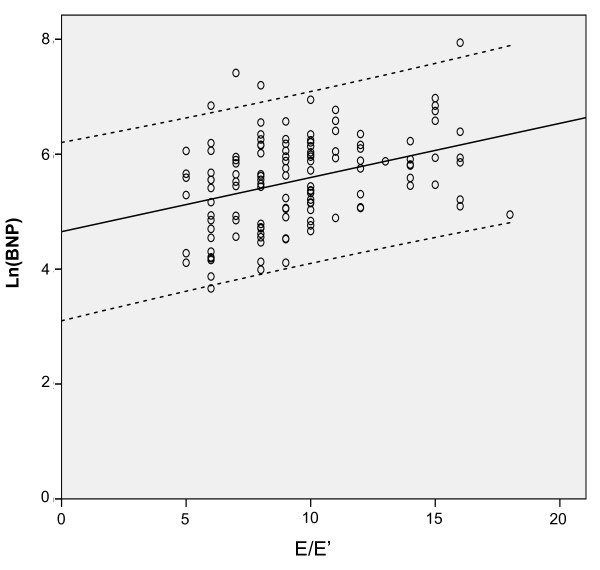
**Correlation analysis of ln(BNP) vs E/E' ratio**. Circles represent individual values of brain natriuretic peptide. The black line represents the linear regression curve fit and the dotted lines its 95% confidence interval.

**Figure 2:**
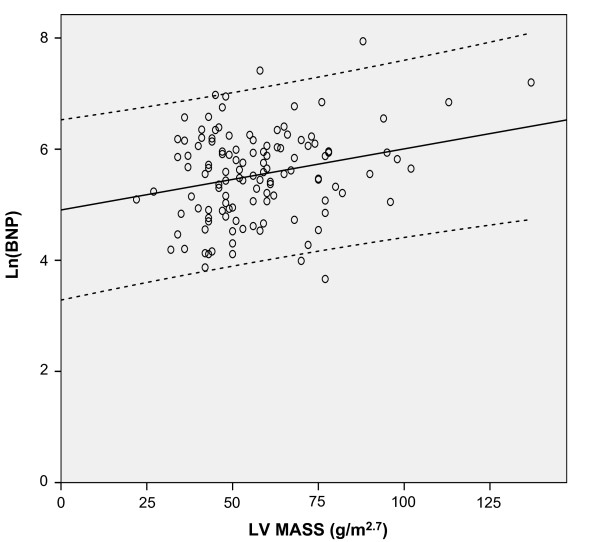
**Correlation analysis of ln(BNP) vs left ventricular (LV) mass**. Circles represent individual values of brain natriuretic peptide. The black line represents the linear regression curve fit and the dotted lines its 95% confidence interval.

**Figure 3:**
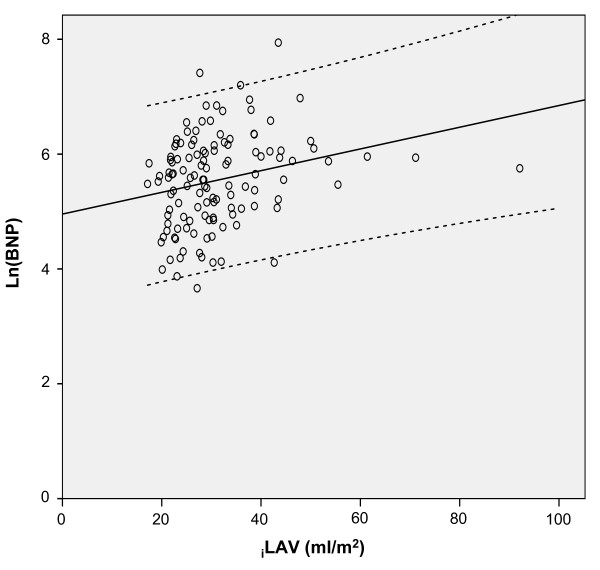
**Correlation analysis of ln(BNP) vs indexed left atrial volume (_i_LAV)**. Circles represent individual values of brain natriuretic peptide. The black line represents the linear regression curve fit and the dotted lines its 95% confidence interval.

The mean LVEF was 59 ± 10%; 108 patients (87%) had a LVEF ≥50%. Mean ln(BNP) levels and E/E' were similar in patients with LVEF≥50% compared to patients with LVEF<50% (Table 3). The echocardiographic parameters of the study group are presented in Table 4. There was no correlation between ln(BNP) and LV volumes, LVEF, RWT, E/A, DT. The relationship between ln(BNP) and E/E' values is depicted in Figure 1, showing that BNP is directly related to LV filling pressure (Pearson's coefficient: 0.36, p < 0.001). Overall, patients with low BNP levels had low values of E/E' ratio [see Additional file 1], while patients with high BNP levels had the highest E/E' ratios [see Additional file 2]. A similar correlation was found between ln(BNP) and LV mass (Pearson's coefficient 0.25, p = 0.006) (Figure 2), and, albeit weaker, between ln(BNP) and _i_LAV (Pearson's coefficient 0.2, p = 0.026) (Figure 3). However, when entered into a multiple regression model, age and E/E' remained the only independent predictors of BNP levels (Table 5).

**Table 3:** Mean value of ln(BNP) and E/E' according to the presence or absence of left ventricular systolic dysfunction.

	LVEF <50%	LVEF ≥50%	
	**(n = 16)**	**(n = 108)**	

			

Ln(BNP)	5.4 ± 0.5	5.5 ± 0.8	n.s.

			

E/E'	9.6 ± 3.7	9.4 ± 2.9	n.s.

**Table 4:** Echocardiographic parameters in the study group.

Parameter	Mean ± SD	Pearson's coefficient	p value
			

LVDV (ml)	80.4 ± 32.3	0.015	0.883

LVSV (ml)	34.8 ± 21.3	0.070	0.507

LVEF (%)	59 ± 10	-0.15	0.11

LV mass (g/m^2.7^)	57.3 ± 18.3	0.25	0.006

RWT	0.48 ± 0.10	0.061	0.50

iLA volume (ml/m^2^)	31.2 ± 10.7	0.2	= 0.026

E/A	1.06 ± 0.34	-0.028	0.772

DT (msec)	203 ± 52	0.093	0.336

E/E'	9.48 ± 3.07	0.36	<0.001

**Table 5:** Multivariable linear regression for ln(BNP) as dependent variable (covariates: E/E', age, LV mass, LVEF, _i_LAV, gender).

Variable	Regression coefficient	95% CI	Standardized coefficient	p value
				

E/E'	0.068	0.016-0.119	0.256	0.011

Age, yrs	0.018	0.003-0.032	0.2220	0.015

LV mass	0.006	-0.001-0.014	0.147	0.101

LVEF	-0.011	-0.025-0.003	-0.025	0.134

_i_LAV	0.003	-0.005-0.011	0.075	0.410

Gender	-0.012	-0.330-0.306	0.007	0.941

Model	R^2 ^= 0.222			<0.001

## Discussion

Elevated BNP levels have been previously reported both prior to and immediately after cardiac surgery. Such findings have been associated with cardiac complications, prolonged hospital stay, and 1-year mortality [8,14,15]. However, the reasons for BNP increase in this clinical setting have not been clarified yet, with BNP inconsistently associated with a variety of factors related to surgery, such as biomarkers of myocardial damage, anaesthesia, sternotomy, etc [16]. Since BNP increase reflects hemodynamic changes, we believe that the ultimate reason for BNP increase in these patients could be the loading changes that occur during and after cardiac surgery. Thus, we hypothesized that elevated LV filling pressures might be the trigger for BNP release.

The main findings of the present study can be summarized as follows:

1. the vast majority of post-cardiac surgery patients show a significant increase in BNP levels;

2. the increase in BNP levels does not correlate with LV systolic function;

3. E/E' ratio, an echocardiographic marker of filling pressures, is strongly associated with elevated BNP levels.

### BNP and ventricular function

In patients with heart failure, plasma BNP levels are inversely related to LV systolic function [17]. In contrast, in our study population we did not find any correlation between BNP levels and systolic LV function as evaluated by echocardiographic ejection fraction. This finding is not surprising, since in the study group, which represents consecutive patients admitted to our Center, the prevalence of LV systolic dysfunction is rather low (only 3 patients had a LVEF <40%). Although our population was not selected, this might not represent the whole spectrum of post cardiac surgery patients.

More recently, in patients with heart failure, an increase in BNP levels has also been related to diastolic (dys)function [4,18]. To this aim, most studies have used conventional echocardiographic parameters, such as mitral inflow diastolic velocities. However, in patients with preserved LV systolic function, i.e. the vast majority of those included in our study, the diagnostic value of these parameters is rather limited and newer echocardiographic modalities are warranted for a comprehensive categorization of the patient's hemodynamic profile. Doppler myocardial velocities at the mitral annulus are widely used for a (relatively) load-independent evaluation of diastolic properties, and left atrial volume is increasingly considered as a long-term biomarker of average LV diastolic pressure [19].

### BNP and newer echocardiographic parameters of diastolic dysfunction

#### E/E' ratio as an index of LV filling pressures

Several studies have evaluated the relationship between BNP levels and the E/E' ratio in different clinical settings (ambulatory patients,[20] heart failure[21]), with consistent results that demonstrate a relationship between BNP levels and echocardiographic indices of LV filling pressures. The findings of the present study obtained in post-cardiac surgery patients are in line with those from previous reports in different clinical settings: we observed a significant correlation between BNP levels and the E/E' ratio, indicating elevated filling pressures as the major determinant of BNP release.

#### Left atrium as an index of chronic diastolic dysfunction

During diastole, the left atrium is directly exposed to LV pressures. Abnormal LV diastolic function causes an elevation of filling pressures, which may lead to left atrial dilatation over time. In the present study, _i_LAV is associated with BNP values, however when entered into a multivariable regression analysis this parameter did not retain its statistical power as predictor of BNP levels.

### Additional factors affecting BNP levels

Age, female gender, and renal failure are all variables related to BNP levels [22,23]. In our study, a statistically significant direct relationship between BNP levels and patient age was observed. In contrast, the difference in BNP values according to renal function, although present, was not significant perhaps because of the small sample size of our study population and the exclusion of patients on haemodialysis.

### Study limitations

There are several limitations of our study. First, BNP levels before cardiac surgery were not sampled. Thus, we cannot exclude that at least in some patients BNP levels had been elevated before surgery. Secondly, we did not perform any invasive hemodynamic tests to evaluate the relationship between hemodynamic status and BNP levels. Previous studies have already shown a close correlation between BNP levels and LV end-diastolic pressure. Thirdly, we did not evaluate the impact of cardioplegic arrest on BNP levels. Finally, follow-up data that may show if elevated BNP levels are associated with worse clinical outcome are lacking. Although length in-hospital stay was independent from BNP levels, the potential role of BNP levels for long-term prognostication of post cardiac surgery patients remains to be assessed.

## Conclusion

What is, then, the clinical relevance of this study? We believe that early after cardiac surgery an increase in LV filling pressure is very likely to occur, independent from LV systolic function, and is a trigger for a significant BNP release. In this clinical setting Doppler echocardiogram is a good alternative to BNP samples for assessing LV filling pressures, and E/E' in particular is a practical and more convenient parameter for detecting and monitoring haemodynamic imbalance.

## Competing interests

The authors declare that they have no competing interests.

## Authors' contributions

AS designed the study, interpreted the data and wrote the manuscript. EC participated in the design of the study, interpreted the data and drafted the manuscript. MP analysed the data and revised the manuscript critically. GP analysed the data and revised the manuscript critically. AP and EA reviewed the echocardiograms, performed the measurements and revised the manuscript critically. SLC performed the statistical analysis and revised the manuscript critically. SB revised the manuscript critically and his criticism was very helpful. WAAM revised the manuscript critically and his criticism was very helpful. All Authors read and approved the final manuscript.

## Supplementary Material

Additional file 1 **Echocardiographic data of a patient with normal BNP**. The data provided represent two-dimensional images and Doppler samples of a 65-year old man after coronary artery bypass graft. In this patient, E/E' was 5.7, consistent with normal LV filling pressures, and BNP levels were normal (64 pg/ml). Click here for file

Additional file 2 **Echocardiographic data of a patient with high BNP**. The data provided represent two-dimensional images and Doppler samples of a 64-year old man after surgery for ascending aorta aneurysm. In this case, E/E' was 16.4, suggesting elevated LV filling pressures, and BNP levels were significantly elevated (855 pg/ml). Click here for file
