# Supplementary figures and images for: Relationship between B-type natriuretic peptide levels and echocardiographic indices of left ventricular filling pressures in post-cardiac surgery patients
Source: Cardiovasc Ultrasound. 2009 Oct 28;7:49. doi: 10.1186/1476-7120-7-49 (PMC2779791; doi:10.1186/1476-7120-7-49)

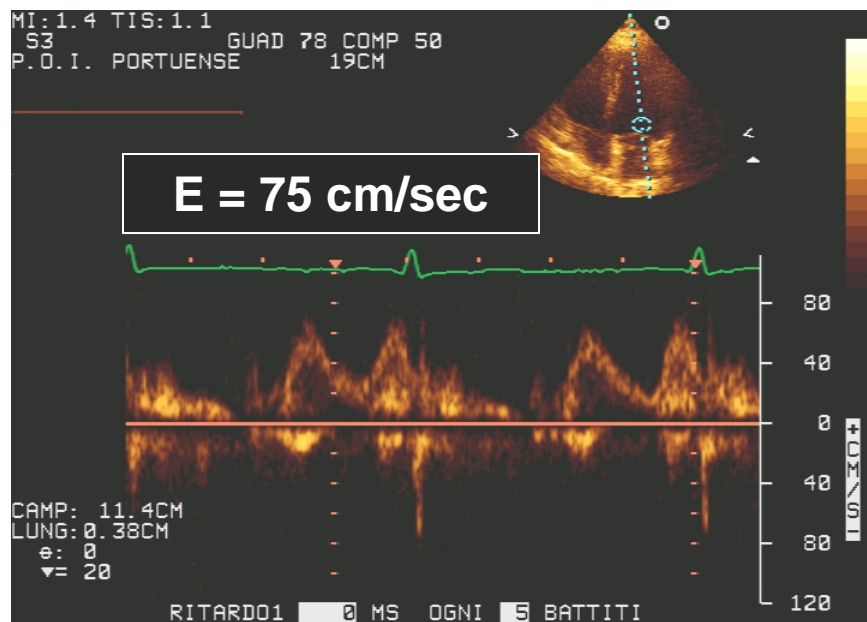

**Mitral flow**

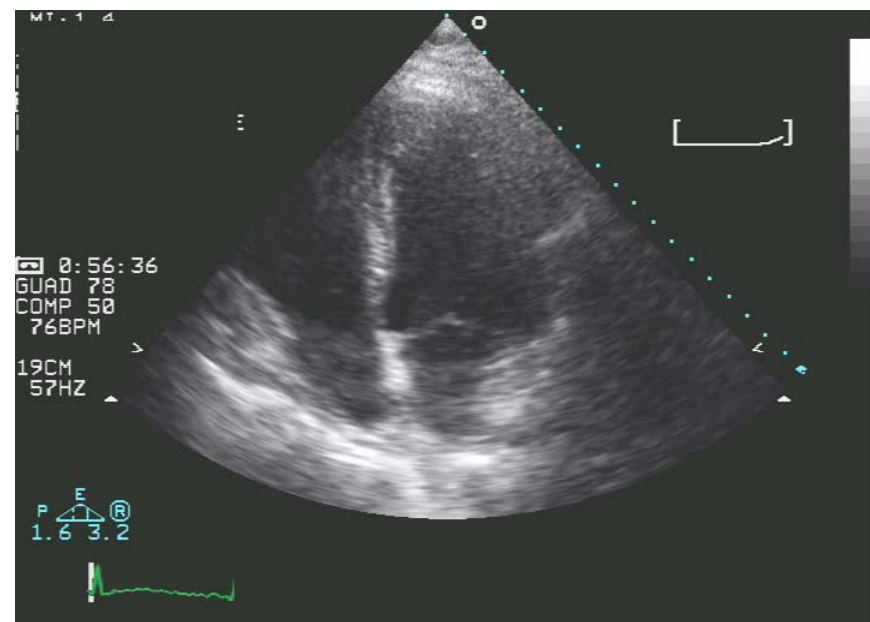

**4-Ch**

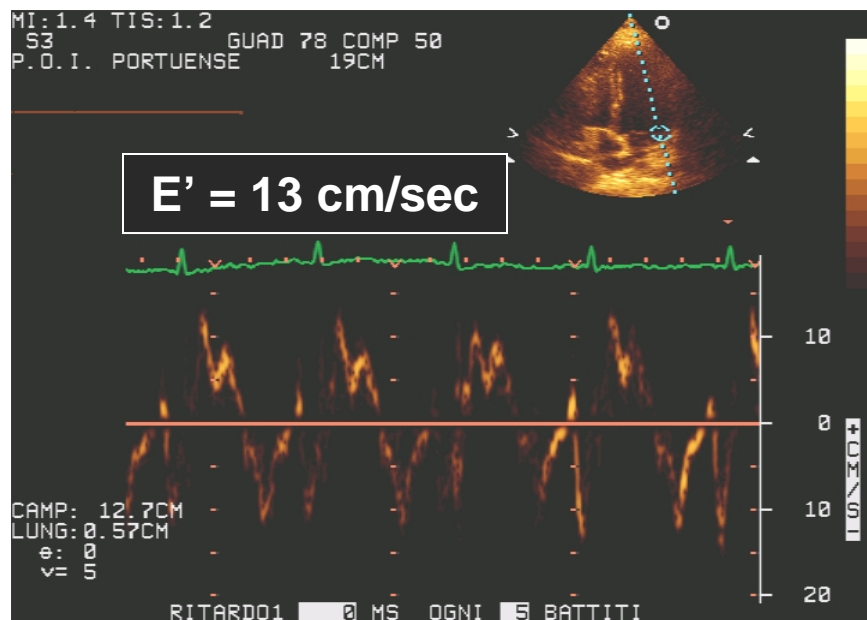

**Mitral annulus - E/E' = 5.7**

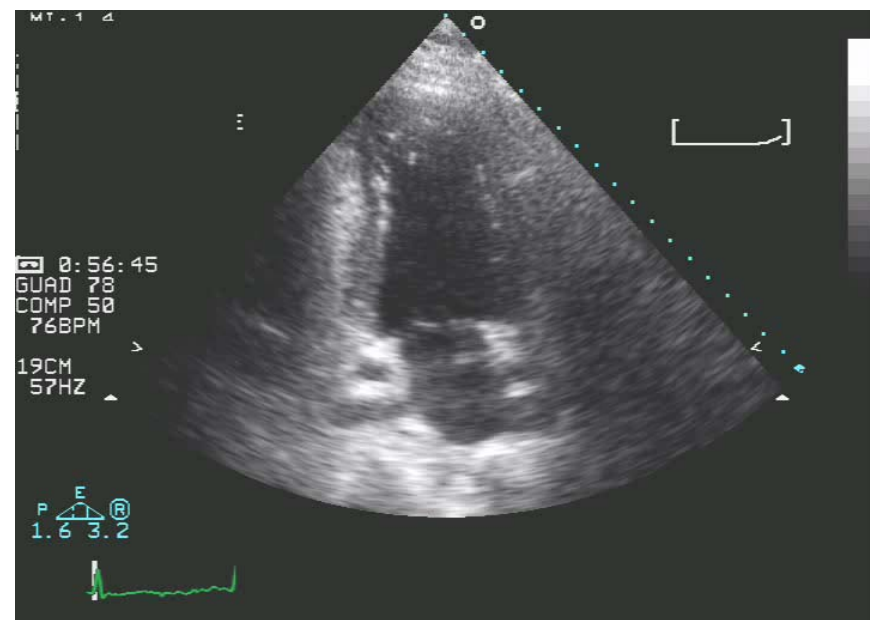

**2-Ch**

Supplement: Additional file 1 — Echocardiographic data of a patient with normal BNP. The data provided represent two-dimensional images and Doppler samples of a 65-year old man after coronary artery bypass graft. In this patient, E/E' was 5.7, consistent with normal LV filling pressures, and BNP levels were normal (64 pg/ml). [file 1476-7120-7-49-S1.pdf]

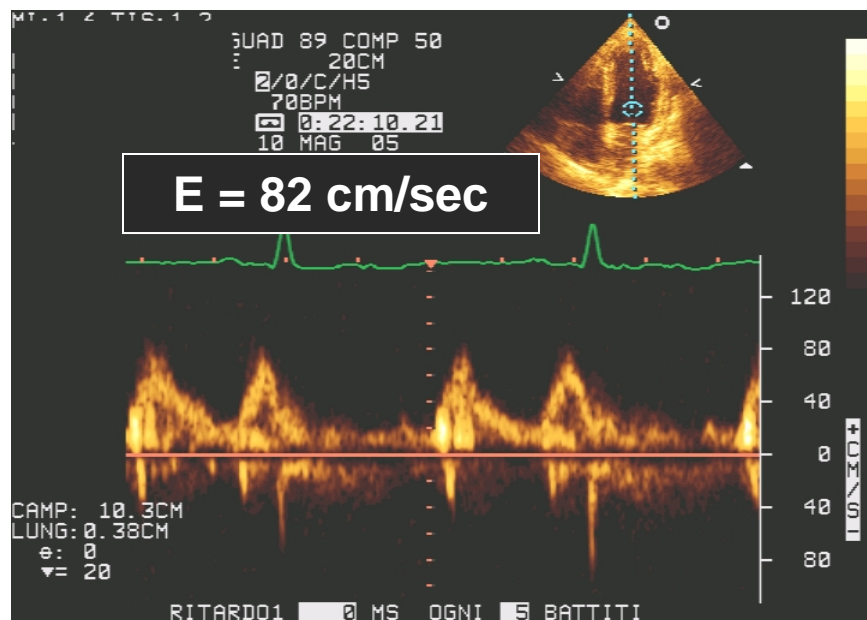

**Mitral flow**

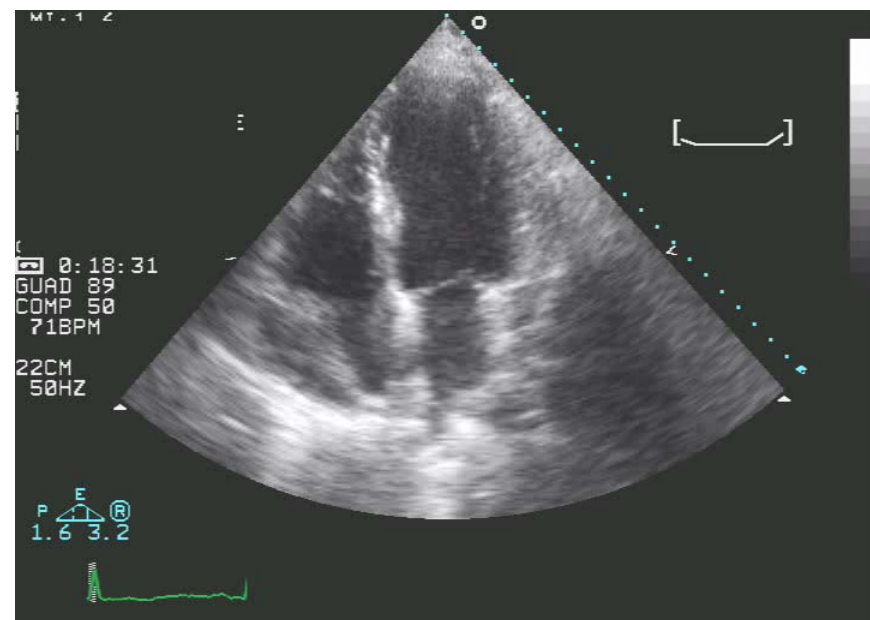

**4-Ch**

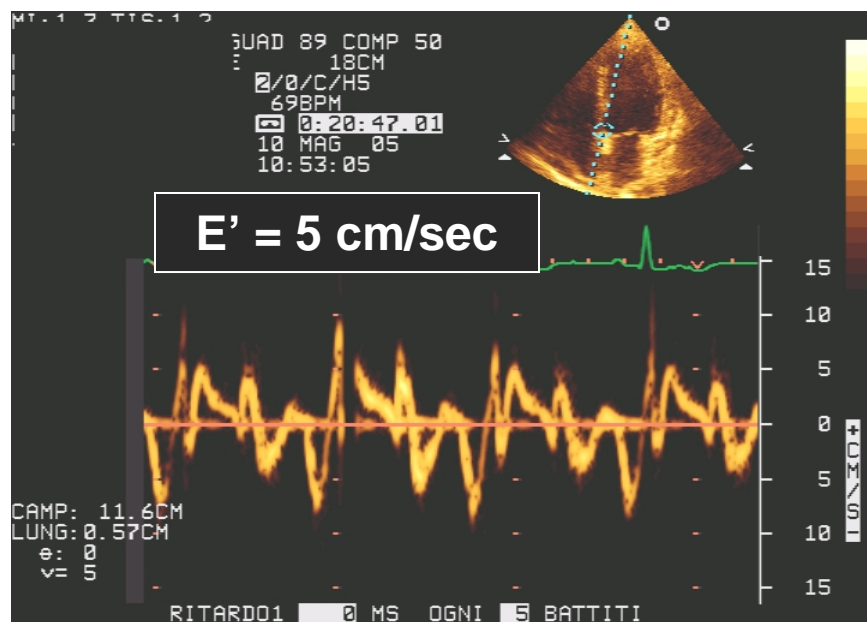

**Mitral annulus - E/E' = 16.4**

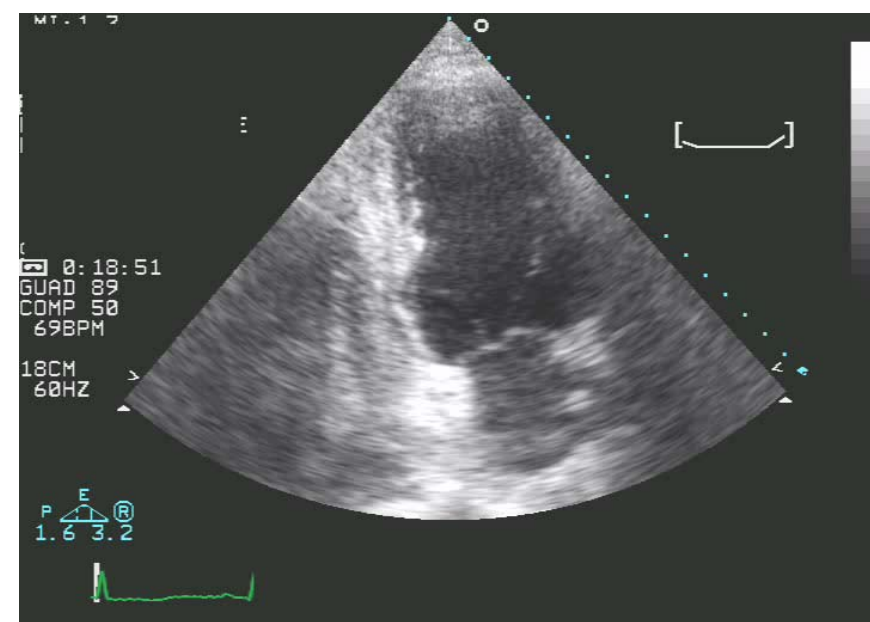

**2-Ch**

Supplement: Additional file 2 — Echocardiographic data of a patient with high BNP. The data provided represent two-dimensional images and Doppler samples of a 64-year old man after surgery for ascending aorta aneurysm. In this case, E/E' was 16.4, suggesting elevated LV filling pressures, and BNP levels were significantly elevated (855 pg/ml). [file 1476-7120-7-49-S2.pdf]
